# Optical tissue measurements of invasive carcinoma and ductal carcinoma in situ for surgical guidance

**DOI:** 10.1186/s13058-021-01436-5

**Published:** 2021-05-22

**Authors:** Lisanne L. de Boer, Esther Kho, Koen K. Van de Vijver, Marie-Jeanne T. F. D. Vranken Peeters, Frederieke van Duijnhoven, Benno H. W. Hendriks, Henricus J. C. M. Sterenborg, Theo J. M. Ruers

**Affiliations:** 1 Department of Surgery, Netherlands Cancer Institute-Antoni van Leeuwenhoek Hospital, Postbus 90203, 1006 Amsterdam, BE Netherlands; 2 Department of Pathology, Ghent University Hospital, and Cancer Research Institute Ghent (CRIG), Ghent University, Ghent, Belgium; 3 Philips Research, In-body Systems Group, Eindhoven, Netherlands; 4 Biomechanical Engineering Department, Delft University of Technology, Delft, The Netherlands; 5 Department of Biomedical Engineering and Physics, Amsterdam University Medical Center, Amsterdam, the Netherlands; 6 Faculty of Science and Technology, University of Twente, Enschede, the Netherlands

**Keywords:** Image-guided surgery, Tissue characterization, Optical spectroscopy, Breast-conserving surgery, Ductal carcinoma in situ (DCIS), Invasive carcinoma (IC)

## Abstract

**Background:**

Although the incidence of positive resection margins in breast-conserving surgery has decreased, both incomplete resection and unnecessary large resections still occur. This is especially the case in the surgical treatment of ductal carcinoma in situ (DCIS). Diffuse reflectance spectroscopy (DRS), an optical technology based on light tissue interactions, can potentially characterize tissue during surgery thereby guiding the surgeon intraoperatively. DRS has shown to be able to discriminate pure healthy breast tissue from pure invasive carcinoma (IC) but limited research has been done on (1) the actual optical characteristics of DCIS and (2) the ability of DRS to characterize measurements that are a mixture of tissue types.

**Methods:**

In this study, DRS spectra were acquired from 107 breast specimens from 107 patients with proven IC and/or DCIS (1488 measurement locations). With a generalized estimating equation model, the differences between the DRS spectra of locations with DCIS and IC and only healthy tissue were compared to see if there were significant differences between these spectra. Subsequently, different classification models were developed to be able to predict if the DRS spectrum of a measurement location represented a measurement location with “healthy” or “malignant” tissue. In the development and testing of the models, different definitions for “healthy” and “malignant” were used. This allowed varying the level of homogeneity in the train and test data.

**Results:**

It was found that the optical characteristics of IC and DCIS were similar. Regarding the classification of tissue with a mixture of tissue types, it was found that using mixed measurement locations in the development of the classification models did not tremendously improve the accuracy of the classification of other measurement locations with a mixture of tissue types. The evaluated classification models were able to classify measurement locations with > 5% malignant cells with a Matthews correlation coefficient of 0.41 or 0.40. Some models showed better sensitivity whereas others had better specificity.

**Conclusion:**

The results suggest that DRS has the potential to detect malignant tissue, including DCIS, in healthy breast tissue and could thus be helpful for surgical guidance.

**Supplementary Information:**

The online version contains supplementary material available at 10.1186/s13058-021-01436-5.

## Background

The incidence of positive resection margins in breast cancer surgery has decreased over time due to improved imaging and a changed definition of a “positive” resection margin [1, 2]. Despite this decrease, re-excisions because of positive resection margins still occur frequently, especially in patients with ductal carcinoma in situ (DCIS) [3]. In this patient group, tumor-positive resection margins are reported in up to 35% of the patients [4–8]. In addition to this, excision volumes are not always matching with optimal resection volumes. Unnecessarily large volumes of healthy tissue might be excised while clear margins are not assured [9–11]. This is important as the quantity of resected tissue negatively impacts the cosmetic result [12–17]. Approximately 30% of patients treated for breast cancer report a poor or fair cosmetic result after breast-conserving surgery [18, 19]. Patients that experience a poor outcome are likely to remain unsatisfied with the outcome 2 to 6 years after surgery and the percentage of women that report poor esthetic outcomes increases in the long-term follow-up [18]. Furthermore, the poor cosmetic outcome was associated with lower quality of life [14, 20, 21] which is persistent over time [20].

Providing intraoperative tissue characterization to the surgeon could improve the outcome of breast-conserving surgery, with fewer positive resection margins and better cosmetic results, and consequently reduce healthcare costs [22]. Diffuse reflectance spectroscopy (DRS) is an optical technology that seems to be an outstanding candidate to fulfill this unmet clinical need as this technology is based on endogenous tissue contrast, is non-destructive, can be performed in real time, and does not require highly skilled personnel. For a DRS measurement, light from a broadband light source is transmitted into the tissue via a fiber integrated into a fiber-optic probe that is brought in contact with the tissue. Inside the tissue, the light is absorbed and scattered after which it is collected with another fiber in the fiber-optic probe. This fiber is connected to a spectrometer that produces a spectrum of the diffusely reflected light (DRS measurement). The amount of absorption and scattering of the light in the tissue depends on the composition and morphology of the tissue.

DRS has shown to have the potential to discriminate healthy breast tissue from invasive carcinoma (IC). However, compared to IC, the number of DRS measurements of DCIS reported in the literature is small. DCIS can be a precursor stage for invasive carcinoma and is structurally different from IC. DCIS is non-invasive and confined to the milk ducts whereas in the case of IC, the basal membrane has disappeared and cancer has become invasive. These conditions are also different from healthy tissue in which these ducts are still present and tend to be smaller and not filled with cells. Since the morphology of these tissue types is different, this might also result in differences in the measured diffuse reflectance.

Furthermore, evaluation of DRS has been focused on discriminating “pure” healthy tissue from “pure” IC whereas little is known on the performance of DRS measurements in more heterogeneous tissue containing multiple tissue types. Investigating both issues is crucial before the technology can be implemented in clinical practice. In this study, we aim to address these issues by acquiring optical DRS measurements from ex vivo breast specimens with a fiber-optic probe. To determine the value of DRS for the detection of DCIS, the measurements of IC, DCIS, and healthy tissue are compared based on spectral features derived from the spectra. Spectral features that show potential for discriminating healthy tissue from malignant tissue are subsequently used to develop classification algorithms. In the development of the classification algorithms, different definitions for “healthy” and “malignant” were used to evaluate which definition would result in optimal classification of inhomogeneous measurement locations with a combination of different tissue types. Finally, these classification models are evaluated with a completely independent dataset to assess the accuracy for detecting DRS measurement locations including some malignant tissue (IC or DCIS).

## Methods

### Tissue slices

Fiber-optic diffuse reflectance spectra were acquired from fresh tissue slices originating from resection specimens of patients who had undergone breast surgery for proven IC and/or DCIS in the Netherlands Cancer Institute-Antoni van Leeuwenhoek Hospital. The study was approved by the hospital. According to Dutch guidelines, no informed consent had to be acquired which was confirmed by the local ethics committee.

Immediately after surgery, when the surgeon was done performing the resection and filling in the paperwork, the specimen was brought from the OR to the pathology department. At the pathology department, the specimens were processed according to standard protocol. A pathologist inked each of the margins with a specific color and sometimes the tissue was frozen to facilitate cutting of the specimen. Subsequently, the specimen was cut in a bread-loafed manner into slices with a thickness between 3 and 10 mm. One of the slices with, if present, macroscopically visible tumor tissue, was used for measuring. Per specimen, one slice was available for measurements.

### Measurement setup

The measurement setup consisted of a fiber-optic probe and two spectrometers (DU420A-BRDD & DU492A-1.7, Andor Technology, Belfast, Northern Ireland) together covering the wavelength range between 400 and 1600 nm. The blunt fiber-optic probe was attached to the spectrometers. Inside the fiber-optic probe, three different fibers with a core diameter of 200 μm were integrated. One of the fibers was used for illuminating the tissue, the other two fibers were used for collecting the light. These were placed next to each other at a distance of 1 mm away from the illuminating fiber and were each connected to one of the two spectrometers.

Before each measuring session, a calibration was performed using a calibration cap with a Spectralon reference standard at the bottom. A similar measurement setup has been used previously and was described in detail elsewhere [23, 24]. Compared to this setup, the setup used in the current study has a smaller distance between the illuminating and collecting fiber (1 mm versus 2.48 mm). Due to the smaller fiber separation distance, the measurement volume will be smaller and spectra will be influenced more by scattering interactions relative to the number of absorption events. This smaller fiber distance makes this data also less suitable for analysis with an analytical model based on diffusion theory as was done in these publications. Therefore, in this current study, the data were analyzed by comparing the measured intensity and using machine learning algorithms.

For measuring, a custom-made grid was placed on top of the tissue slice that defined the measurement locations. The fiber-optic probe was fixed in this grid during the acquisition of the data. The process of correlating the location of a measurement with the histopathology and estimating the composition of the measurement location is explained in detail in Additional file 1. In short, a registration was made between the RGB image of the tissue without grid and the hematoxylin and eosin (HE) stained section of the tissue slice. The HE sections were evaluated by the pathologist who annotated the different tissue types and specifically estimated the percentage of malignant cells (if present) in a measurement location. Also, a registration was made between the RGB image of the tissue with the grid and the RGB image of the tissue without the grid. This step allowed to display the measurement locations in the RGB without the grid. Combining this RGB image with measurement locations with the labeled HE section allowed estimating the tissue composition in each measurement location. These pathology results were used to label the measurement locations for the development of the classification models.

Pathology ink that was present on the ex vivo specimens affected the visual wavelengths. Therefore, only the spectrum between 850 and 1600 nm was used in the analysis. In total, 80 features were extracted from all the spectra for the analysis (Additional files 2, 3, 4, 5 and 6). These spectral features included slopes between wavelength pairs and spectral features that were derived from the local minima and local maxima of the spectra.

### IC vs DCIS vs healthy

To assess the optical characteristics of DCIS, the spectra of locations with only DCIS were compared with the spectra of locations with only IC. Also, the spectra of both these malignant tissue type locations were separately compared with the spectra of locations with only healthy tissue. This way, it is possible to assess the differences between IC and DCIS as well as the optical contrast between the malignant tissue types and healthy tissue. For comparing DRS spectra of measurement locations with DCIS to DRS spectra from measurement locations with IC, measurement locations were selected that contained either IC or DCIS in combination with surrounding connective tissue. Connective tissue was allowed to be present in these malignant measurement locations as there were no measurement locations that consisted of only IC cells or DCIS cells without any connective tissue. For the healthy tissue measurement locations, all measurement locations that consisted of only fat and/or connective tissue were selected.

A generalized estimating equation (GEE) model was generated with each spectral feature to assess if a spectral feature was statistically different in the comparison between two tissue types. The analysis was performed with GEE models as these are suitable for modeling correlated data by assessing associations between measurements and covariates while taking into account the inter-patient correlation between measurements. For describing the variance and covariance between repeated measurements, an equicorrelated structure was used for all GEE models. In each model, the tissue type (‘IC’/‘DCIS’/‘healthy’) was provided as a covariate as well as the percentage of connective tissue at a measurement location. All analyses were performed in SPSS (IBM SPSS Statistics 25, Armonk, New York, USA). A spectral feature was considered significant when the *p* value lower than 0.05 was calculated.

### Classification model development

Classification models were developed to predict if the DRS spectrum from a measurement location represented a “healthy” or “malignant” measurement location. The hypothesis was that for classifying mixed measurement locations a model that was developed with measurement locations with a mixture of tissue types would result in improved accuracy. To this end, the definitions of “healthy” and “malignant” were modified for developing different classification models. A measurement location was labeled “malignant” based on two criteria: (1) the presence of malignant cells (IC or DCIS), and (2) the percentage of fat in the measurement location does not exceed Thres_maxfat_. This percentage of fat was set between 0 and 100% at 10% intervals. The percentage of fat was varied in the definition of “malignant” as fat is the predominant tissue type in healthy breast tissue. Measurement locations were labeled as “healthy” if they met the following criteria: (1) the absence of malignant cells (IC or DCIS), and (2) the percentage of connective tissue present in the measurement volume does not exceed Thres_maxcon_. Thres_maxcon_ was set between 0 and 100% at 10% intervals. For the definition of “healthy,” the percentage of connective was varied as connective tissue is often present in tumor tissue. Measurement locations that did not meet the definition of “healthy” or “malignant” were labeled as “mixed.”

For each combination of Thres_maxfat_ and Thres_maxcon_, a classification model was developed with measurement locations that were “healthy” or “malignant” according to the definitions. Thus in total, 121 models were developed. A linear support vector machine (SVM) was used in each of the models to discriminate between the two classes. The SVM was weighted to compensate for the imbalance between the number of “healthy” and “malignant” measurements.

**Fig. 1:**
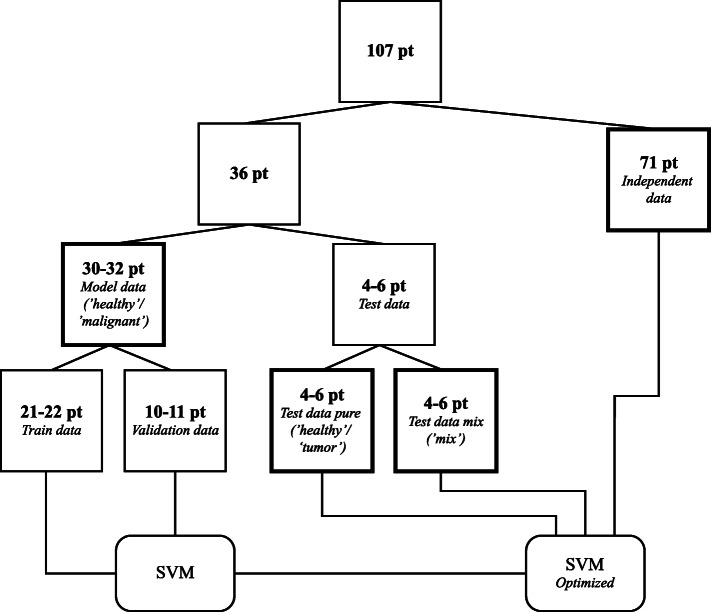
Flowchart of the splitting of data into three subsets. The data was split into test data (pure and mix), model data, and independent data. The model data and test data were used to develop a classification model based on a linear, weighted support vector machine (SVM), and test this classification model. The independent data was classified with the developed classification model to evaluate the performance of the classification model on a separate dataset

To avoid bias in the model, development and assessment of the performance different datasets were formed for training, validating, and testing the models. This was done by splitting the dataset of 107 patients into several subsets (Fig. 1). The data of 36 patients (~ 33%) was used to both develop and test a classification model. This dataset was again split into two sets of data via 8-fold cross-validation; (1) data for developing the classification model; the *Model data* (“healthy” and “malignant” measurement locations), and (2) data for testing the model once it had been optimized; the *Test data pure* (“healthy” and “malignant” measurement locations) and *Test data mix* (“mix” measurement locations). Via 3-fold cross-validation, the *Model data* was further split into the *Train data* (“healthy” and “malignant” measurement locations) and *Validation data* (‘healthy’ and ‘malignant’ measurement locations) which was used for optimizing the classification model. The definition of “healthy” and “malignant” depended on Thres_maxfat_ and Thres_maxcon_ and as a consequence measurement locations could have different labels in the development of the different models.

The splitting of the data was based on the patient number to ensure that the data used for training and testing the different models always originated from the same patient for each of the 121 models.

The second group of 71 patients formed the *independent data* (“healthy,” “malignant,” and “mix” measurement locations) which was completely independent of the data used for the development of the models. This data was used to further assess the performance of the classification models.

### Performance of the classification models

#### Test data pure and test data mix

To assess the accuracy of the classification models for classifying the DRS measurements, first, the *test data pure* was classified. The performance of the individual classification models was assessed by calculating the Matthews correlation coefficient (MCC). To test for overfitting, also the *model data* was classified with the classification models and compared to the MCC of the *test data pure*. Subsequently, classification models with high MCCs were selected and used to classify the *test data mix* (measurement locations labeled as “mix”) and an MCC was calculated. This enabled us to investigate the performance of the classification models for classifying measurement locations with a mixture of tissue types.

#### Independent data

The *independent data* consisted of data from patients that were not used for developing or testing the classification models. To evaluate the performance of the classification models on this dataset, the classification output was compared to the composition of the measurement location. Subsequently, also the MCC, as well as the sensitivity, and specificity were calculated for detecting all measurement locations with 5–40% malignant cells to relate the performance of the classification model to the clinical setting.

## Results

Fiber-optic diffuse reflectance spectra of 1488 measurement locations from 107 resection specimens of 107 patients were available for analysis. Fat, connective tissue, IC, and DCIS were present in respectively 90.5%, 86.9%, 14.6%, and 8% of the measurement locations.

In Table 1, the characteristics of the patients are displayed. The mean age was 56.3 years (± 11.5 years). Of the 107 patients, 58 were postmenopausal (54.2%), 33 patients were premenopausal (9.3%), 10 patients were perimenopausal (9.3%), and menopausal status was unknown for 6 patients (5.6%). In total, 44 patients had received a preoperative treatment with either chemotherapy (30 patients, 28%) or hormonal therapy (14 patients, 13.1%).

**Table 1:** Patient characteristics

Age	
Mean	56.3 years
Standard deviation	11.5 years
Menopausal status	
Premenopausal	33 patients (30.8%)
Perimenopausal	10 patients (9.3%)
Postmenopausal	58 patients (54.2%)
Unknown	6 patients (5.6%)
Preoperative treatment	
Chemotherapy	30 patients (28%)
Hormonal therapy	14 patients (13.1%)

### IC vs DCIS vs healthy

**Fig. 2:**
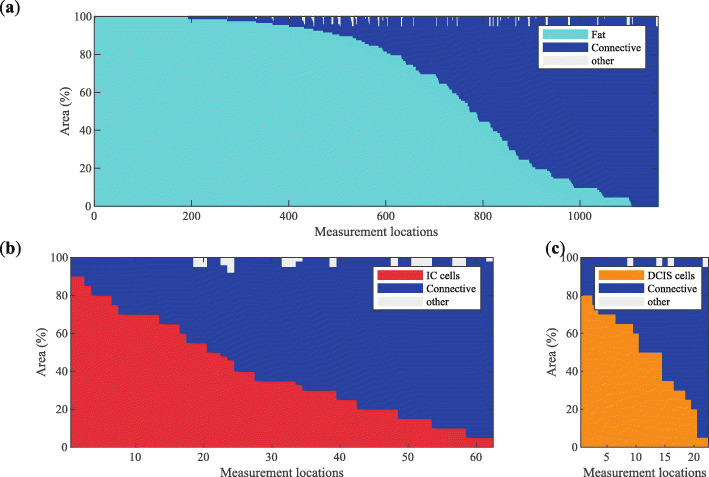
Composition of the locations selected for comparing the differences between measurements of different tissue types. The three groups that were defined were healthy tissue measurement locations (**a**), measurement locations of IC (**b**), and measurement locations of DCIS (**c**)

From the total dataset of 1488 measurement locations, the measurement locations with either IC, DCIS, or healthy tissue only were selected (1244 measurement locations) to compare how the spectral features differed between these groups of measurement locations. The composition of these selected measurement locations is given in Fig. 2.

As can be seen in Fig. 2a, some of the healthy measurement locations consisted of 100% fat or connective tissue. On the other hand, there are no measurement locations that consist of only malignant cells (Fig. 2b, c). In the case of IC, the highest percentage of IC cells is 90%, and for DCIS measurement locations, this is 80%.

**Fig. 3:**
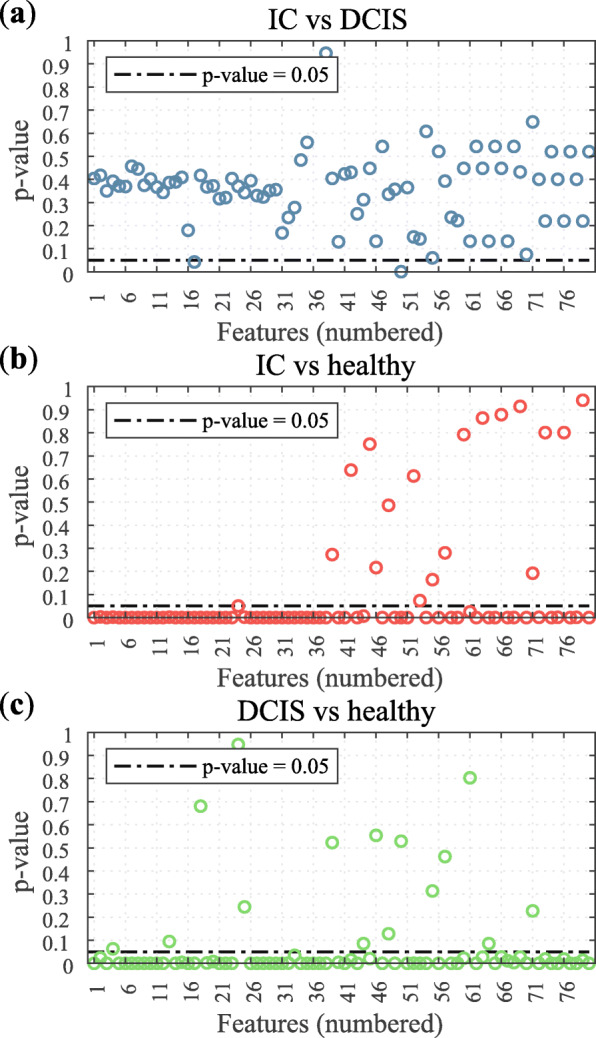
*P* values of each feature for comparing measurement locations with IC vs measurement locations with DCIS (**a**), measurement locations with IC vs measurement locations with only healthy tissue types (**b**), and measurement locations with DCIS vs measurement locations with only healthy tissue types (**c**)

The GEE results of the majority of spectral features did not have *p* values below 0.05 and are therefore not significantly different between the measurement locations with IC and the measurement locations with DCIS (Fig. 3). Only two features were significantly different with a *p* value below 0.05; (1) the slope between 999 nm and 1034 nm and (2) the wavelength of the inflection point on the right side of the local minimum at 1205 nm (see Additional file 7).

On the other hand, the vast majority of spectral features was significantly different for IC measurement locations in comparison to healthy tissue measurement locations (62 of 80 features), and for DCIS measurement locations in comparison to healthy tissue measurement locations (65 of 80 features). Altogether, in 75% (60/80) of the features, the calculated *p* values for both the IC and DCIS comparison with healthy tissue measurement locations show the same result. The fact that the results were consistent for IC and DCIS confirmed that the measured DRS spectra of these malignant tissue types are similar. Thus, it was concluded that the optical characteristics of IC and DCIS are the same.

### Performance of classification models

**Fig. 4:**
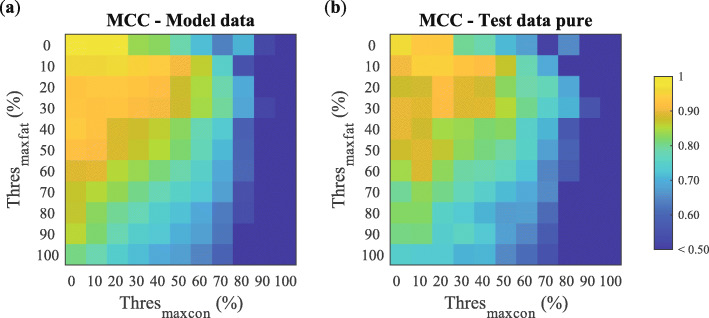
Mean MCC of model data (**a**) and test data pure (**b**) for all 121 models. Each model was developed with a different Thres_maxfat_ and Thres_maxcon_ that determined which measurement locations were labelled “healthy” or “malignant”

In total, 121 classification models (11 possibilities for Thres_maxcon_ multiplied by 11 possibilities for Thres_maxfat_) were developed and evaluated on the classification performance. For the model development, the number of features was further reduced to a set of 16 features by performing a floating feature search (See Additional file 8). Figure 4a shows the MCC on the *model data*, and Fig. 4b shows the MCC on the *test data pure* for all models. In general, for all models, the MCC of the *model data* is slightly better than the MCC of the *test data pure*. The differences between the two are small which indicates that the models are not overfitting. The MCC decreases with an increasing percentage of fat in the measurement locations defined as “malignant” (i.e., an increasing percentage of Thres_maxfat_) and an increasing percentage of connective tissue in the measurement locations defined as “healthy” (i.e., an increasing percentage of Thres_maxcon_).

Regarding the *test data pure*, the performances of the models with a Thres_maxfat_ of 0% and a Thres_maxcon_ of 0%, 10%, or 20% are excellent with MCCs of 0.97, 0.93, and 0.93 respectively. With a Thres_maxfat_ of 10%, the performances on this dataset of the models with a Thres_maxcon_ of 0%, 10%, and 20% are still very good with MCCs ranging between 0.91 and 0.95.

Four models marking the corners of the box between a Thres_maxfat_ of 0 to 10% and a Thres_maxcon_ of 0 to 20% in Fig. 4b were selected to assess the ability of the classification models to correctly classify measurement locations with a mixture of tissue types. The selected models were as follows: (1) Thres_maxfat_ 0%/Thres_maxcon_ 0% (Model I), (2) Thres_maxfat_ 0%/Thres_maxcon_ 20% (Model II), (3) Thres_maxfat_ 10%/Thres_maxcon_ 0% (Model III), and (4) Thres_maxfat_ 10%/Thres_maxcon_ 20% (Model IV). These models were selected as they have similar performance but different definitions of “healthy” and “malignant.” Model I is based on the most homogeneous data whereas in the three other models more fat tissue and/or connective tissue was allowed in the definition of “healthy” and “malignant.”

#### Test data pure and test data mix

**Fig. 5:**
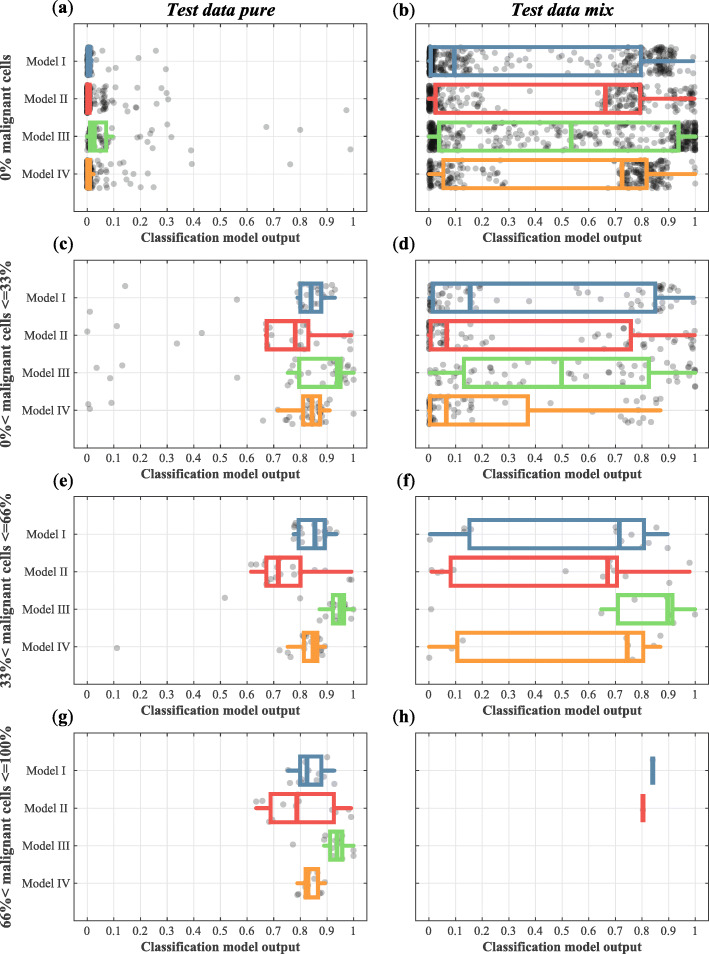
Results on the classification of “healthy” and “malignant” locations from the test data pure (left) and “mix” locations from test data mix (right). In each graph, the *x*-axis represents the output of the classification model. A value between 0 and 0.5 is considered “healthy” by the classification models and a value between 0.5 and 1 is considered “malignant” by the classification model. The graphs (**a**, **b**) show the classification results of measurements with 0% malignant cells, (**c**, **d**) the results of measurement locations in which 1 to 33% of the volume was comprised of malignant cells, in (**e**, **f**) this percentage ranges from 34 to 66%, and in (**g**, **h**) measurement locations contained more than 66% malignant cells

Figure 5 shows the results of the four selected classification models on the *test data pure* (left) and *test data mix* (right). Regarding the *test data pure*, for the measurement locations with 0% malignant cells, the mean output of the four classification models is close to 0 which represents classification as “healthy” (Fig. 5a). For the three categories with malignant cells (Fig. 5c, e and f), the mean output of all the classification models is close to 1 and thus these measurement locations were classified as “malignant.” Hence, the classification accuracy of all models is similar and excellent for classifying measurements without malignant cells as “healthy” and classifying measurement locations with malignant cells as “malignant.”

The results on the *test data mix* show that for measurement locations with 0% malignant cells (Fig. 5b) the mean output of Model I was below 0.5, and for Model II, Model III, and Model IV, the median was higher than 0.5. Hence, for these three models, a substantial group of measurement locations without malignant cells was classified as “malignant.”

Classification of measurement locations with a low percentage of malignant cells (≤ 33%) seems difficult for all models but one. Only Model III shows a mean classification model output of 0.5 for this category (Fig. 5d). For classification of measurement locations with higher percentages of malignant cells, the median of the classification model output for the *test data mix* dataset increased with an increasing percentage of malignant cells in the measurement location (Fig. 5d, f, h). In the category with > 66% malignant cells (Fig. 5h), only limited data were available, however, and no measurement locations could be classified by Model III and Model IV. The reason for this is that more measurement locations are labeled as “healthy” and “tumor” and are therefore used in the development of the classification model. For all of these models, the standard deviations are large.

### Composition of independent data

**Fig. 6:**
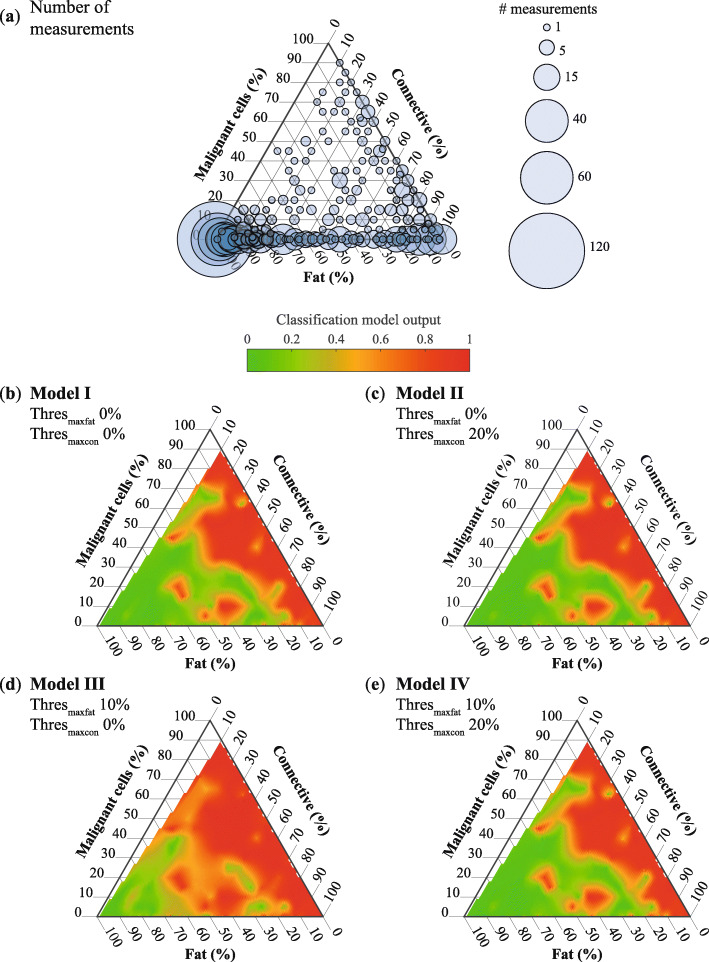
Ternary plots of independent data. Each point in a plot sums to a total of 100%. The three corners of the plot represent measurement locations of 100% malignant cells, 100% connective tissue, and 100% fat tissue. Points within the triangle represent mixtures of these substances. For each measurement location, the percentage of one of these substances decreases linearly with increasing distance from the corner that represents 100% of the substances. **a** Composition of the measurement locations. The size of the dots is proportional to the number of measurement locations with that specific tissue composition. **b**–**e** Classification model output of Model I–Model IV

The classification models were used on the *independent data* of 71 patients. The data of these patients were not used in the development of the classification models. The measurement locations in this dataset were both measurement locations with only one tissue type or a mixture of tissue types and should be a representation of the composition of breast tissue in general. The tissue composition of the measurement locations in the *independent data* is depicted in Fig. 6a. The large circles in the left corner of the graph in Fig. 6a indicate that the majority of the measurement locations had a high percentage of fat. Furthermore, the majority of the circles are located at the lower border of the triangle. Hence, a large portion of the measurement locations did not have any malignant cells but was composed of fat and connective tissue (88% of measurement locations). Except for the 100% fat measurement locations, there are no circles depicted on the left border of the triangle implying that there was always some connective tissue present in those measurement locations.

### Classification of independent dataset

Figure 6b–e depicts the interpolated classification results by each of the four models of the dataset of *independent data* in triangle plots. Comparing the performance of Model I to Model II, the difference is that more connective tissue (up to 20%) is allowed in the definition of “healthy.” However, there is hardly any effect of this change in definition in the results on the *independent data*. The effect of changing the definition of “malignant” by allowing more fat tissue (up to 10%) can be seen by comparing Model I with Model III. Especially, the locations in the middle of the ternplot, which are locations with a mixture of tissue types, are more often classified as “malignant.” All measurement locations with more than 40% of malignant cells have a classification model output that suggests tumor. Lastly, in Model IV, both up to 20% of connective tissue is allowed in the definition of “healthy” and up to 10% of fat tissue is allowed in the definition of “malignant.” The classification model output of this model is comparable to the classification model output of Model I and Model II.

Finally, the MCC, sensitivity, and specificity were calculated for all four classification models for the detection of measurement locations with a certain percentage of malignant cells. The results are shown in Table 2.

**Table 2:** Performance of the classification models for the detection of measurement locations with a different threshold for the percentages of malignant cells in the locations labeled as “malignant”

	Percentage malignant cells	Model I	Model II	Model III	Model IV
MCC	5%	0.41	0.41	0.40	0.41
	10%	0.39	0.39	0.37	0.39
	15%	0.40	0.40	0.36	0.40
	20%	0.37	0.37	0.33	0.37
	25%	0.36	0.36	0.32	0.36
	30%	0.35	0.35	0.30	0.35
	35%	0.31	0.31	0.28	0.31
	40%	0.31	0.31	0.28	0.31
Sensitivity	5%	0.62	0.62	0.79	0.62
	10%	0.66	0.66	0.82	0.66
	15%	0.71	0.71	0.86	0.71
	20%	0.73	0.73	0.85	0.73
	25%	0.75	0.75	0.88	0.75
	30%	0.79	0.79	0.90	0.79
	35%	0.77	0.77	0.91	0.77
	40%	0.80	0.80	0.96	0.80
Specificity	5%	0.84	0.84	0.71	0.84
	10%	0.82	0.82	0.70	0.82
	15%	0.82	0.82	0.69	0.82
	20%	0.81	0.81	0.68	0.81
	25%	0.80	0.80	0.67	0.80
	30%	0.80	0.80	0.67	0.80
	35%	0.79	0.79	0.66	0.79
	40%	0.79	0.79	0.66	0.79

Model I, Model II, and Model IV show the same performance and have the highest MCC of 0.41 for the lowest percentage of malignant cells (5%). However, for this percentage of malignant cells, the MCC of Model III is almost similar to the other three models. With an increasing percentage of malignant cells, the MCC decreases for all four models.

For measurement locations with 5% or more malignant cells, Model I, Model II, and Model IV have a higher specificity and a lower sensitivity whereas Model III has a higher sensitivity and lower specificity. With an increasing percentage of malignant cells, the sensitivity increases for all models. For Model III, the sensitivity reaches 0.96 for locations with more than 40% malignant cells. Model I, Model II, and Model IV have a sensitivity of 0.80 for this percentage of malignant cells. Besides an increased sensitivity, a decreased specificity is found with increasing percentages of malignant cells. For locations with more than 5% malignant cells, the specificity is 0.84 (Model I, Model II, and Model IV) and 0.71 (Model III). When more than 40% of malignant cells are present, the specificity is 0.66 for Model III, and 0.79 for Model I, Model II, and Model IV.

## Discussion

In this study, we investigated the potential use of DRS for margin assessment during breast-conserving surgery with ex vivo measurements on breast specimens of 107 unique patients. We aimed to assess (1) the optical difference between measurements of DCIS and IC and (2) the classification of measurements that contain a mixture of tissue types.

First, the optical differences between measurement locations with DCIS and measurement locations with IC were analyzed by comparing the spectral features of the measurements of both groups. The vast majority of spectral features were not significantly different DCIS measurements compared to the IC measurements. Furthermore, 60 out of the total 80 spectral features were significantly different in the comparison of the malignant tissue types (both IC and DCIS) in comparison with the healthy tissue measurements. It was concluded that DCIS and IC have similar optical characteristics and for this reason, for the remaining analyses DCIS and IC were assessed jointly as “malignant cells.” There have been a few reports on DRS measurements of DCIS specifically [25–28]. In these papers, measurements were acquired over a smaller wavelength range, typically only enclosing the visual wavelengths, and often the analysis was performed with optical parameters (i.e., blood content, reduced scattering) that were derived from the measured spectrum. Comparing our measurements with the previously published work is therefore not possible. The main value of our work is that we have gathered a large database of DCIS measurements and that we have shown that DCIS can be detected by DRS. This is of critical importance when the technique is used for evaluating resection margins.

The second topic of this research was the classification of measurements in heterogeneous tissue consisting of a combination of malignant cells and healthy tissue such as fat and connective tissue. We assessed if developing classification models with DRS measurements from a mixture of tissue types would improve the performance for classification of measurement locations with a mixture of tissue types. To this end, first, the models were tested with the *test data pure*. The performance of the models on this data was comparable to results from previous studies [26, 29–31]. Four classification models with high MCCs (> 0.91) on the *test data pure* dataset were used to also classify other datasets with measurement locations that contain a mixture of tissue types. The classification of the *test data mix* was less accurate compared to the *test data pure*, probably because the measurements in the *test data mix* were more heterogeneous compared to the *test data pure*. Model III showed a higher classification model output for the measurement locations with up to 33% malignant cells in comparison to the other three models. This model is thus more sensitive for the correct classification of the measurement locations with a low percentage of malignant cells than the other models. This also suggests that allowing more fat tissue in the definition of “malignant” can help in detecting the measurement locations with a small portion of fat tissue combined with malignant cells. Allowing more connective tissue in the definition of “healthy” did not help in improving the correct classification of healthy measurement locations that could have some connective tissue. An explanation could be that to some extent the connective tissue, that is always present around the malignant cells, is used by the classification models for discriminating “healthy” from “malignant.” It should also be noted that the standard deviation is large especially for the measurement locations without or with lower percentages of malignant cells. This could suggest that there might be subgroups in each category that are either easy or difficult to classify correctly.

The four selected classification models were further tested with the *independent data* which was completely independent of the data used for the development of the classification models. An analysis of the composition of the measurement locations in this dataset revealed that the majority of the measurement locations have a large percentage of fat and a limited presence of malignant cells and that connective tissue was present in almost all measurement locations. This is expected as breast tissue consists of mainly fat tissue interlined with some glandular/connective tissue. Comparing the outputs of all four models, three models seem very similar and only Model III shows a different result. The classification model output of Model I and Model II was very comparable which suggests that allowing up to 20% of connective tissue in the definition of “healthy” did not result in improved classification results of measurement locations without malignant cells that contain a mix of fat tissue and connective tissue. This was also seen in the results of the *test data mix*. An explanation for this could be that the majority of measurement locations with some connective tissue but without malignant cells are already classified correctly as “healthy.” On the contrary, allowing up to 10% of fat tissue in the definition of “malignant” (Model III) resulted in a classification model output of > 0.5 for all measurement locations with more than 40% of malignant cells, independent of the amount of fat and connective tissue. Hence, the presence of a little bit of fat in a measurement location does not automatically result in a classification model output of < 0.5 (e.g., “healthy”). The classification model output of Model IV is very comparable to the outputs of Model I and Model II and distinctly different from Model III. The effect of only allowing more fat tissue in the measurement locations defined as “malignant” seems to be overpowered by the effect of allowing more connective tissue in the measurement locations defined as “healthy.” However, although this seems visually the case this is probably a bias related to the way the results are displayed in the ternplots. All the healthy measurement locations without any malignant cells are visualized on the lower border of the ternplot whereas all the measurement locations that contain a percentage of malignant cells are spread over the rest of the pyramid. This makes visualizing the effects on the healthy measurement locations more difficult in this type of plot.

In Table 2, the effect of the percentage of malignant cells on the performance of the classification models is displayed. With regard to an increasing percentage of malignant cells, all models show similar performance in terms of MCC, sensitivity, and specificity. The sensitivity increases, whereas MCC and specificity decrease. Thus, with a higher percentage of malignant cells present in the measurement location, the models are better at detecting these as “malignant.” However, specificity decreases, thus also more locations without malignant cells are classified as malignant. This can be explained as there is always connective tissue present in the measurement locations that contain malignant cells. Therefore, completely separating connective tissue from malignant cells is impossible and all classification models to some extent seem to use the presence of connective tissue as a surrogate for the presence of malignant cells. This result was also found by other groups [32]. Furthermore, measurement locations with a high percentage of malignant cells, a low percentage of connective tissue, combined with fat tissue were rare. For example, only 50 locations (3.4%) consisted of less than 30% connective tissue and more than 5% malignant cells.

When comparing the performances of the models in terms of sensitivity and specificity differences can be seen between Model III and the other three Models. Model III has a higher sensitivity and lower specificity, whereas the other three models have a lower sensitivity and higher specificity. This is independent of the percentage of malignant cells in the measurement locations. Model III is thus better for detecting all locations with malignant cells, but this is achieved at the expense of misclassifying some healthy measurement locations. Which model is optimal for clinical use, or in other words, if higher sensitivity or specificity is desired, should be researched in a large clinical study. In this study, the output of the models should be related to what is considered a positive resection margin according to the pathologist as well as the consequences of possibly resecting some healthy tissue that is incorrectly classified as malignant.

In this research, only the linear and weighted SVM was used for classifying the measurements. This was a deliberate choice as the linear SVM is relatively insensitive for overfitting and proved useful in previous research [29]. Especially since the dataset is imbalanced, overfitting is a potential danger. It was not in the scope of this research to further assess different types of classification models. However, future research could be directed towards other classification models, which in potency could yield better results.

The feature extraction and selection method used in this research was not pulled from literature but designed by the authors. The slopes, local minima, and local maxima were all extracted with an algorithm from the mean spectra of the “pure” tissue types (e.g., fat, connective, IC, and DCIS). There was an imbalance in the number of spectra used for calculating the mean for each of the tissue types. Extending the number of pure measurements, especially for the tissue classes with a limited number of spectra, could result in a different extraction of features. Once extracted, the features were selected based on the results of the GEE analysis and the floating feature search. No other feature selection methods were evaluated in this research; however, potentially, a different feature selection method could lead to a better performance of the classification model.

The majority of the publications using DRS for margin assessment evaluated the entire margin by using multiple probes and classifying an entire margin as opposed to a single measurement [33, 34]. In the latter case, spatial information is absent, and a fiber-optic probe measurement is one single spectrum that is the sum of the diffuse reflectance of multiple tissue types present in the probed volume. Thus, assessing the influence of mixed tissue is more important in this case. The number of publications that assessed point measurements of measurement locations with a mixture of tissue types is limited. A study by Kennedy et al. evaluated the margin in a point-based method but excluded the mixed tissue locations because the fractional composition of the locations was not specified by histopathology [25]. A publication by Pappo did include locations with a combination of tumor tissue and healthy tissue [35]. In this paper, the MarginProbe is used similarly to the point measurements in our study. The reported sensitivity and specificity for locations with more than 75% tumor tissue were 100% and 87% respectively. However, including the mixed measurement locations, their sensitivity and specificity dropped both to 70%.

To assess the clinical value of DRS, the percentage of malignant cells should be related to the volume of malignant cells that is present in a measurement location in the case of a positive resection margin. Besides, it is important to recognize that the definition of a positive resection margin has shifted over the years and is still under debate [36]. Furthermore, there is a difference between the definition of a positive resection margin for DCIS [37], and the definition of a positive resection margin for IC [38]. Although both definitions have shifted towards a more liberal definition for DCIS, this is still more stringent than for IC. As discriminating the two tissue types with DRS seems impossible, this might have implications for what is detected by DRS as a positive resection margin. Importantly, there is ongoing controversy around the current definition of a positive resection margin for DCIS and this could further shift towards an even more liberal definition in the future [5, 39–42]. There are some indications that a less stringent interpretation of a positive resection margin is safe for DCIS as long as post-operative radiotherapy is not omitted [43].

Even though the number of positive resection margins in breast-conserving surgery for IC and/or DCIS decreased over time, there is a lot to be gained by providing the surgeon with additional information to decreased excised tissue volumes. This was shown in studies in which intraoperative ultrasound was used for margin assessment [44, 45]. However, this technology has not emerged widely in clinical practice probably because it requires additional skills from the surgeon and intense collaboration between the radiology and surgical department [46, 47]. On top of that, it is not suitable for the detection of DCIS as ultrasound imaging has limited sensitivity for this tissue type [48].

## Conclusions

This study investigated the potential of DRS for intraoperative resection margin assessment. First, we found that except for two spectral features, all other 78 spectral features were not significantly different between IC and DCIS. It was concluded that because of this similarity in optical characteristics DCIS can also be detected by DRS. Secondly, developing different classification models with different definitions of “healthy” and “malignant” did not make a huge impact on the classification performance of measurements with a mixture of tissue types. When considering all measurement locations with 5% or more malignant cells as malignant, the MCC of all models was similar (0.40 or 0.41). However, sensitivity and specificity did vary between the models independent of the percentage of malignant cells that was present. Model III had a higher sensitivity and lower specificity than the other three models (Model I, Model II, and Model IV) which had a lower sensitivity and higher specificity. These results should be related to what is considered a positive resection margin and if a classification model with higher sensitivity or higher specificity is desired. Future research should thus be directed to investigating if the accuracy found in this research is sufficient for the intraoperative detection of malignant tissue and can thus improve the surgical treatment of breast cancer.

## Supplementary Information

**Additional file 1.** Method for estimating the tissue composition of the measurement locations. Figure that explains how the HE section and the corresponding annotations were correlated to the optical measurement locations, and how subsequently the composition of the measurement locations was estimated.

**Additional file 2.** Mean DRS spectra and histopathology examples of ‘Fat’, ‘Connective’, ‘IC’, and ‘DCIS’ that were used for extracting the spectral features. This figure displays the mean spectra of the four tissue types and the standard deviation, as well as examples of the corresponding HE samples.

**Additional file 3.** Method of extracting spectral features based on the slope of the mean spectrum. This file describes how for each comparison of two tissue types differences in slope between all possible combinations of wavelengths were assessed.

**Additional file 4.** List of slopes derived from comparing the DRS spectra of different combinations of tissue types. The file lists the complete set of slopes that were found in the analysis and from which comparison of two tissue types these slopes originated.

**Additional file 5.** Method of extracting spectral features based on local minima and local maxima in the mean spectra of Fat, Connective, IC, and DCIS. The figure explains the method for extracting spectral features that are related to the local minima and local maxima in the mean spectra of ‘Fat’, ‘Connective’, ‘IC’, and ‘DCIS’.

**Additional file 6.** List of spectral features derived from the local minima and maxima of Fat, Connective, IC, and DCIS. This table contains a complete list of the local minima and local maxima that were used for extracting spectral features. The table also shows from which mean spectra of a tissue type the local minima and local maxima originated.

**Additional file 7.** Results of the GEE analysis of all spectral features. The table lists all p-values of the spectral features from the GEE-analysis.

**Additional file 8.** Selection of feature set with a floating search. The table lists the set of features that is selected after the floating feature search.

## Data Availability

The datasets used and/or analyzed during the current study are available from the corresponding author on reasonable request.

## Abbreviations

DCIS: Ductal carcinoma in situ
DRS: Diffuse reflectance spectroscopy
IC: Invasive carcinoma
GEE: Generalized estimating equation
MCC: Matthews correlation coefficient
