# Supplementary material for: Optical tissue measurements of invasive carcinoma and ductal carcinoma in situ for surgical guidance
Source: Breast Cancer Res. 2021 May 22;23:59. doi: 10.1186/s13058-021-01436-5 (PMC8141169; doi:10.1186/s13058-021-01436-5)
Supplement: Supplementary file 1 — Additional file 1. Method for estimating the tissue composition of the measurement locations. Figure that explains how the HE section and the corresponding annotations were correlated to the optical measurement locations, and how subsequently the composition of the measurement locations was estimated. [file 13058_2021_1436_MOESM1_ESM.docx]

## Additional file 1

_
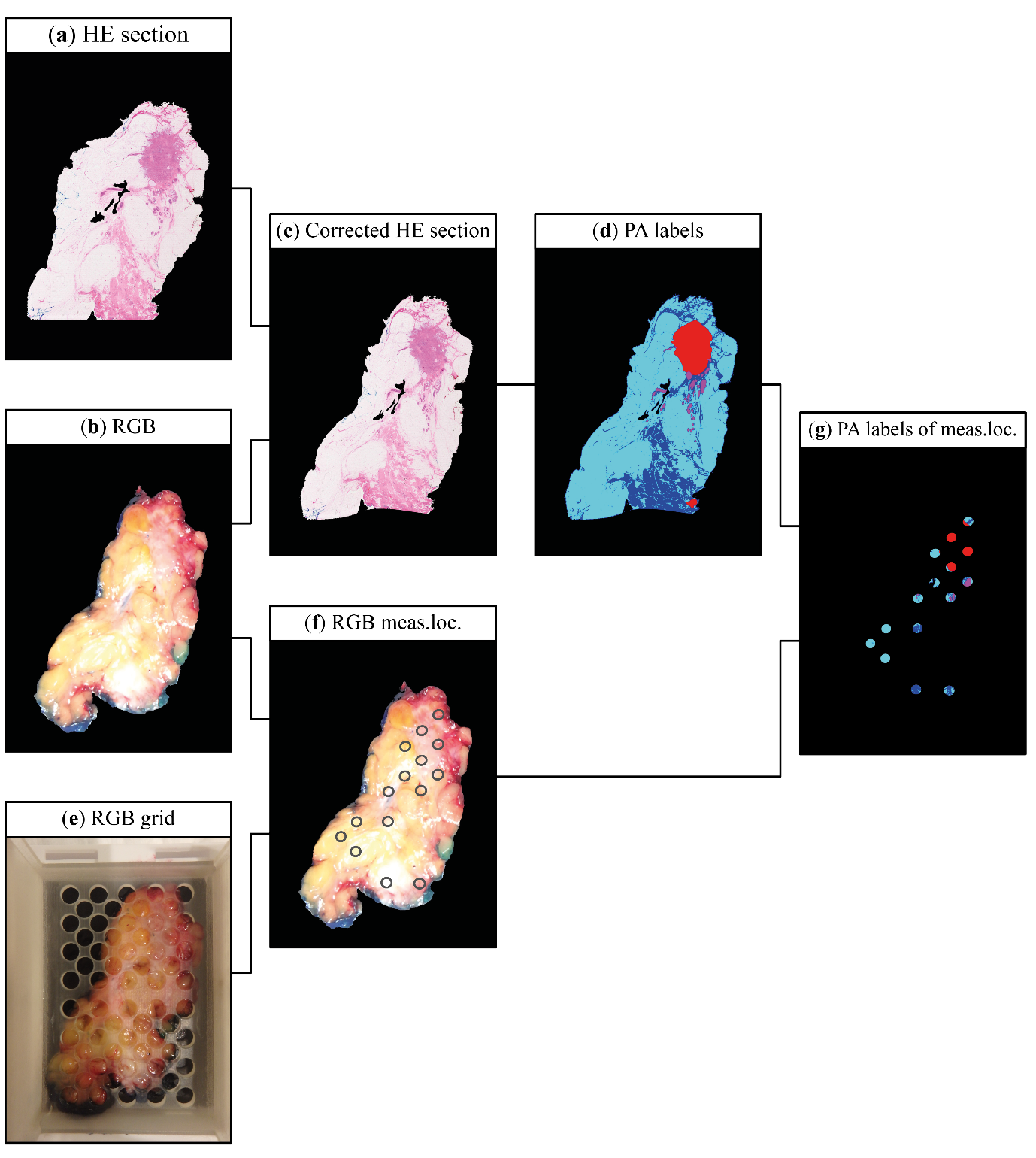
_

**Additional file 1. Method for estimating the tissue composition of the measurement locations.**

First, the image of the HE (hematoxylin and eosin) stained section (**a**) was registered to an overview RGB image (**b**) of the tissue in the macrocassette. This registration allowed to correct for tissue deformation introduced when the tissue was processed by the pathology department. The result was the HE section that is corrected for tissue deformations (**c**). A pathologist assessed each HE section, and circumscribed areas with IC, DCIS, and glandular ducts. The other tissue types, connective tissue and fat tissue stained respectively pink or white in the HE section, and these were annotated automatically by digital thresholding the image. The glandular ducts were included in the connective tissue class. The final result was an image in which each pixel has a histopathology label; ‘IC’, ‘DCIS’, ‘Connective’, or ‘Fat’ (**d**).

Furthermore, A second RGB image of the tissue in the macrocasette with the grid on top of the tissue (**e**) was registered to the overview RGB image (**b**). This registration allowed retrieving the locations on the tissue where the probe was placed during measurements (**f**). Combining the retrieved measurement locations (defined as a circle with a 2 mm cross-section) (**f**) with the image with the labeled pixels provided the pathology labels for each of the measurement locations (**g**). The procedure for correlating the measurements to the histopathology was described in more detail elsewhere.(1) Additionally, for all measurement locations that contained IC or DCIS, the pathologist estimated the percentage of malignant cells in each of those measurement locations. The final result was a score of the percentage of fat, connective tissue, and malignant cells (IC or DCIS) in each of the measurement locations.

1. De Boer LL, Kho E, Nijkamp J, Van de Vijver KK, Sterenborg HJCM, Ter Beek LC, et al. A method for co-registration of optical measurements of breast tissue with histopathology: the importance of accounting for tissue deformations. Journal of Biomedical Optics. 2019;24(7):075002.
