# Supplementary material for: Optical tissue measurements of invasive carcinoma and ductal carcinoma in situ for surgical guidance
Source: Breast Cancer Res. 2021 May 22;23:59. doi: 10.1186/s13058-021-01436-5 (PMC8141169; doi:10.1186/s13058-021-01436-5)
Supplement: Supplementary file 2 — Additional file 2. Mean DRS spectra and histopathology examples of ‘Fat’, ‘Connective’, ‘IC’, and ‘DCIS’ that were used for extracting the spectral features. This figure displays the mean spectra of the four tissue types and the standard deviation, as well as examples of the corresponding HE samples. [file 13058_2021_1436_MOESM2_ESM.docx]

## Additional file 2

**
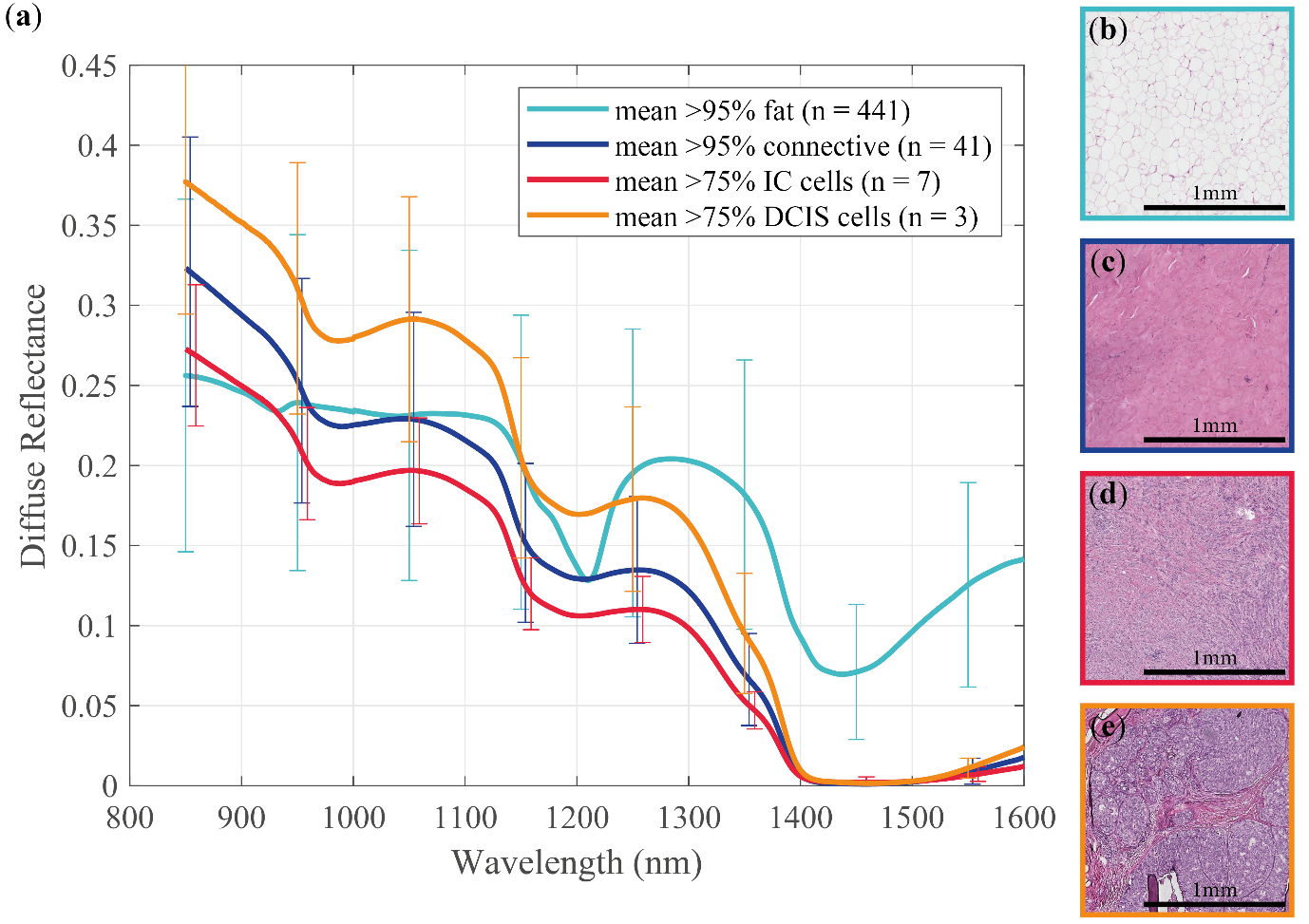
**

**Additional file 2. Mean DRS spectra and histopathology examples of ‘Fat’, ‘Connective’, ‘IC’, and ‘DCIS’ used for extracting the spectral features.**

In the analysis spectral data was reduced to a limited number of spectral features. For extracting these spectral features first the mean DRS spectrum of all tissue types present in the measured specimens was calculated. The mean spectrum of ‘Fat’ (percentage fat ≥ 95%), ‘Connective’ (percentage connective ≥ 95%), ‘IC’ (percentage IC cells ≥ 75% & percentage fat = 0), and ‘DCIS’ (percentage DCIS cells ≥ 75% & percentage fat = 0) with standard deviation in error bars are displayed in (**a**). The number between the brackets indicates the number of spectra. (**b**-**e**) Typical examples of fat, connective, IC, and DCIS in sections colored with hematoxylin and eosin (HE) staining.
