# Supplementary material for: Optical tissue measurements of invasive carcinoma and ductal carcinoma in situ for surgical guidance
Source: Breast Cancer Res. 2021 May 22;23:59. doi: 10.1186/s13058-021-01436-5 (PMC8141169; doi:10.1186/s13058-021-01436-5)
Supplement: Supplementary file 3 — Additional file 3. Method of extracting spectral features based on the slope of the mean spectrum. This file describes how for each comparison of two tissue types differences in slope between all possible combinations of wavelengths were assessed. [file 13058_2021_1436_MOESM3_ESM.docx]

## Additional file 3

­
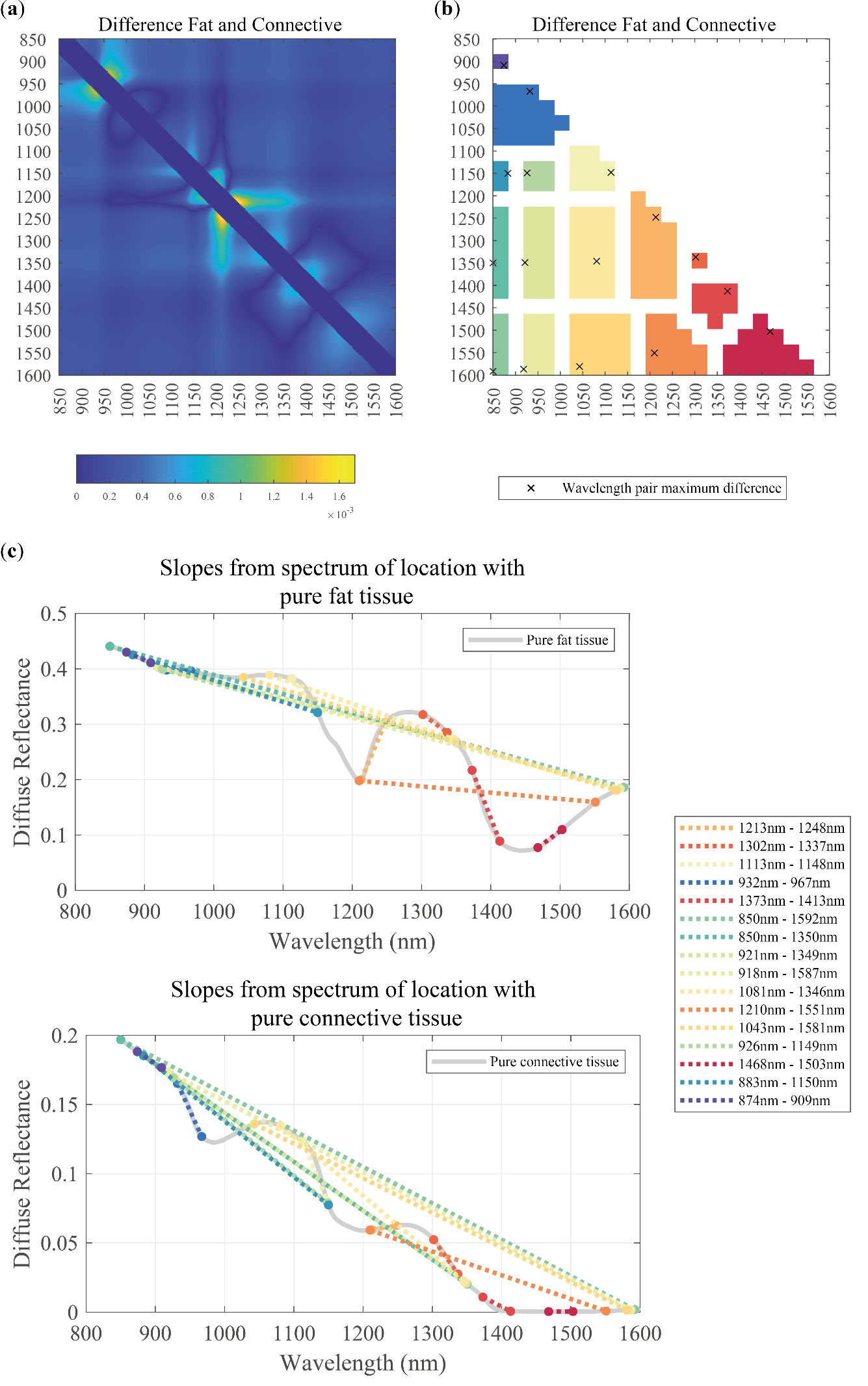


**Additional file 3. Method of extracting spectral features based on the slope of the mean spectrum.** Example of extraction of slopes that were different between the mean spectrum of ‘Fat’ and ‘Connective’. First, we calculated the absolute difference between the slope of the mean fat spectrum and the mean connective spectrum for each pair of wavelengths (ranging between 850 and 1600 nm) (**a**). The distance between two wavelengths in a pair should be at least 35 nm to avoid sensitivity for small intensity changes. Subsequently, by using a watershed method, areas with a similar difference were grouped (**b**). More weight was assigned to wavelength pairs that were further apart. If the start and end wavelengths of two pairs were both within 10 nm from each other, one of the two pairs was excluded. In each area, the wavelength pair with the largest absolute difference is displayed with a black cross (**c**). This procedure was repeated for all combinations of tissue types, thus ‘Fat’ vs ‘IC’, ‘Fat’ vs ‘DCIS’, ‘Connective’ vs ‘IC’, ’Connective’ vs ‘DCIS’, and ‘IC’ vs ‘DCIS’.
