# Supplementary material for: Optical tissue measurements of invasive carcinoma and ductal carcinoma in situ for surgical guidance
Source: Breast Cancer Res. 2021 May 22;23:59. doi: 10.1186/s13058-021-01436-5 (PMC8141169; doi:10.1186/s13058-021-01436-5)
Supplement: Supplementary file 4 — Additional file 4. List of slopes derived from comparing the DRS spectra of different combinations of tissue types. The file lists the complete set of slopes that were found in the analysis and from which comparison of two tissue types these slopes originated. [file 13058_2021_1436_MOESM4_ESM.docx]

**Additional file 4**

| *Slopes* | *F vs C* | *F vs IC* | *F vs DCIS* | *C vs IC* | *C vs DCIS* | *IC vs DCIS* |
| --- | --- | --- | --- | --- | --- | --- |
| 850-1122nm |  |  |  | x |  |  |
| 850-1210nm |  |  |  | x |  |  |
| 850-1350nm | x |  |  |  |  |  |
| 850-1440nm |  |  |  | x |  |  |
| 850-1584nm |  |  | x |  |  |  |
| 850-1592nm / 850-1598nm | x | x |  |  |  |  |
| 861-907nm / 867-905nm |  | x | x |  |  |  |
| 870-1084nm |  |  |  |  | x |  |
| 874-909nm / 875-911nm | x |  |  |  |  | x |
| 883-1149nm / 883-1150nm | x | x |  |  |  |  |
| 918-1587nm / 918-1594nm | x | x |  |  |  |  |
| 921-1349nm / 924-1346nm / 924-1354nm | x | x | x |  |  |  |
| 925-1419nm |  |  |  |  |  | x |
| 926-1149nm / 927-1149nm / 928-1152nm | x | x | x |  |  |  |
| 931-1195nm |  |  |  |  |  | x |
| 932-967nm / 936-971nm | x | x | x |  | x | x |
| 999-1034nm |  |  |  |  | x |  |
| 1024-1059nm |  |  |  | x |  |  |
| 1043-1581nm / 1046-1590nm | x | x |  |  |  |  |
| 1051-1557nm |  |  | x |  |  |  |
| 1081-1346nm / 1082-1343nm | x | x |  |  |  |  |
| 1091-1348nm |  |  | x |  |  |  |
| 1112-1147nm / 1113- 1148nm | x | x |  |  |  |  |
| 1121-1404nm / 1123-1405nm / 1130-1407nm |  |  |  | x | x | x |
| 1125-1160nm / 1127-1162nm / 1134-1169nm |  |  |  | x | x | x |
| 1201-1236nm |  |  |  |  | x |  |
| 1210-1551nm | x |  |  |  |  |  |
| 1210-1561nm |  | x |  |  |  |  |
| 1211-1517nm |  |  | x |  |  |  |
| 1213-1248nm | x | x | x |  |  |  |
| 1214-1339nm |  |  | x |  |  |  |
| 1299-1334nm / 1302-1337nm / 1305-1340nm | x | x | x |  |  |  |
| 1312-1347nm |  |  |  |  | x |  |
| 1363-1398nm |  |  |  | x | x | x |
| 1372-1409nm / 1373-1413nm | x | x |  |  |  |  |
| 1395-1430nm |  |  | x |  |  |  |
| 1467-1502nm / 1468-1503nm | x | x | x |  |  |  |
| 1517-1552nm / 1521-1556nm / 1522-1557nm |  |  |  | x | x | x |
| F = Fat, C = Connective, IC = Invasive Carcinoma, DCIS = Ductal Carcinoma In Situ | | | | | | |

**Additional file 4. List of slopes derived from comparing the DRS spectra of different combinations of tissue types.** In some cases, the slope from one comparison of tissue types showed overlap with the slope from another comparison between two tissue types. For example in the comparison of ‘Fat’ with ‘Connective’ the slope between 850 nm and 1592 nm differed significantly. However, for the comparison of ‘Fat’ with ‘IC’, the slope between 850nm and 1598nm differed significantly. Since the first wavelength was equal for both slopes and the second wavelengths were just 6nm apart these were combined into one spectral feature.
