# Supplementary material for: Optical tissue measurements of invasive carcinoma and ductal carcinoma in situ for surgical guidance
Source: Breast Cancer Res. 2021 May 22;23:59. doi: 10.1186/s13058-021-01436-5 (PMC8141169; doi:10.1186/s13058-021-01436-5)
Supplement: Supplementary file 5 — Additional file 5. Method of extracting spectral features based on local minima and local maxima in the mean spectra of Fat, Connective, IC, and DCIS. The figure explains the method for extracting spectral features that are related to the local minima and local maxima in the mean spectra of ‘Fat’, ‘Connective’, ‘IC’, and ‘DCIS’. [file 13058_2021_1436_MOESM5_ESM.docx]

## Additional file 5


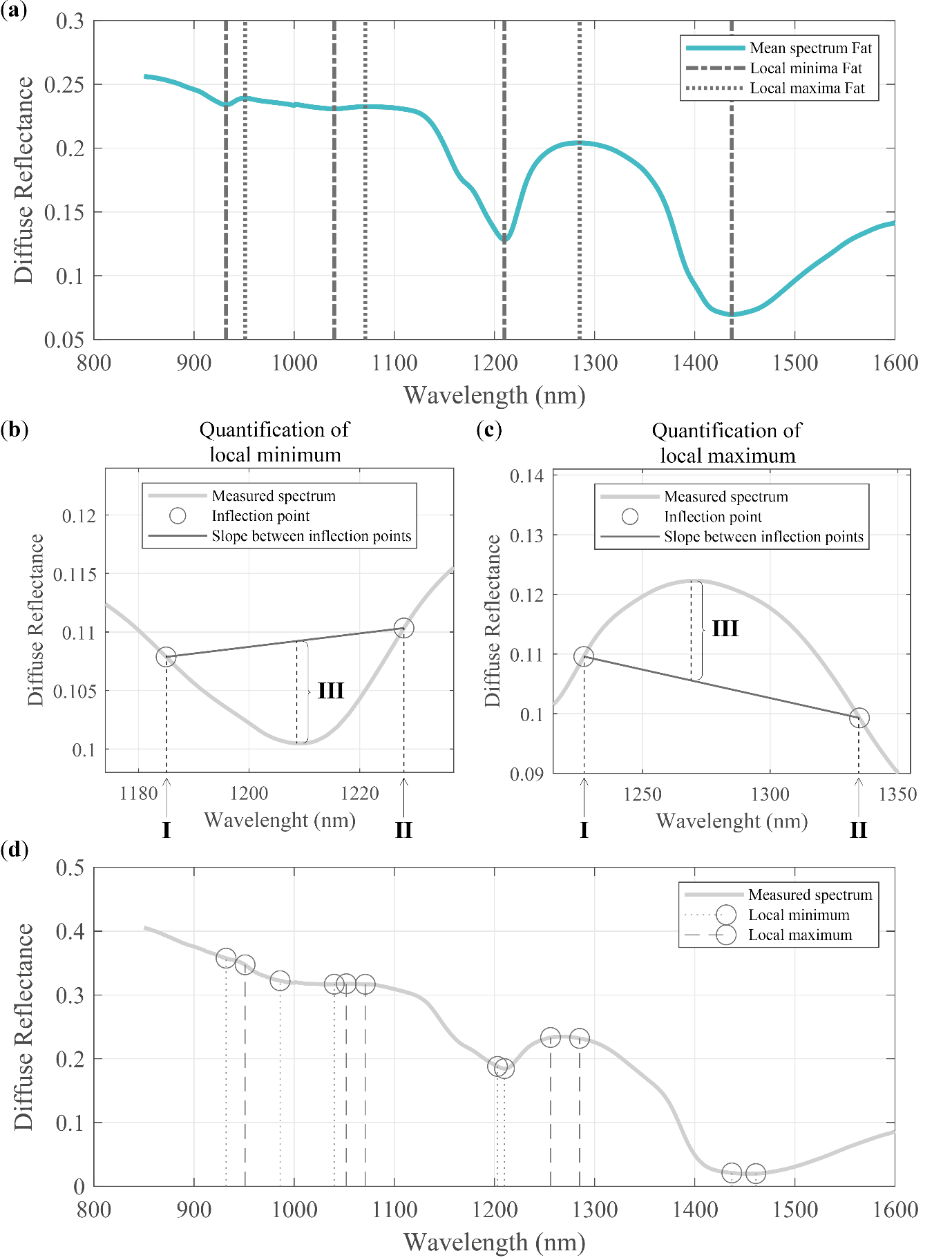


**Additional file 5. Method of extracting spectral features based on local minima and local maxima in the mean spectra of Fat, Connective, IC, and DCIS.** Example of extraction of spectral features related to dips and peaks in the mean spectrum of ‘Fat’. The local minima and maxima were derived from the mean spectrum using the ‘islocalmin’ and ‘islocalmax’ functions of Matlab (**a**). The minimal peak prominence was set to 0.001. K-means clustering was performed to cluster local minima and local maxima that originated from different mean spectra but which were only a few nm apart. For the local minima and local maxima that remained after the clustering the wavelength left and right of the minimum or maximum was determined by searching for the inflection points left and right of the local minimum or local maximum. In total per local minimum (**b**) and local maximum (**c**), three features were derived. The first feature was the wavelength of the inflection point left of the local minimum or local maximum (I). The second feature was the wavelength of the inflection point right of the local minimum or local maximum (II). The last feature was the maximum difference between the slope between feature I and feature II and the measured spectrum (III). This procedure was repeated for the mean spectrum of connective tissue, IC, and DCIS. In Additional File 6 the complete list of features extracted from the local minima and local maxima can be found.
