# Supplementary material for: Optical tissue measurements of invasive carcinoma and ductal carcinoma in situ for surgical guidance
Source: Breast Cancer Res. 2021 May 22;23:59. doi: 10.1186/s13058-021-01436-5 (PMC8141169; doi:10.1186/s13058-021-01436-5)
Supplement: Supplementary file 6 — Additional file 6. List of spectral features derived from the local minima and maxima of Fat, Connective, IC, and DCIS. This table contains a complete list of the local minima and local maxima that were used for extracting spectral features. The table also shows from which mean spectra of a tissue type the local minima and local maxima originated. [file 13058_2021_1436_MOESM6_ESM.docx]

## Additional file 6

|  | Fat | Connective | IC | DCIS |
| --- | --- | --- | --- | --- |
| *Local minima* |  |  |  |  |
| 932 nm | x |  |  |  |
| 987 nm |  | x | x | x |
| 1040 nm | x |  |  |  |
| 1205 nm | x | x | x | x |
| 1437 nm | x |  |  |  |
| 1461 nm |  | x | x | x |
| *Local maxima* |  |  |  |  |
| 951 nm | x |  |  |  |
| 1046 nm |  | x |  |  |
| 1052 nm |  |  | x |  |
| 1054 nm |  |  |  | x |
| 1071 nm | x |  |  |  |
| 1256 nm |  | x | x |  |
| 1259 nm |  |  |  | x |
| 1285 nm | x |  |  |  |

**Additional file 6. List of spectral features derived from the local minima and maxima of Fat, Connective, IC, and DCIS.** Some local minima were present in the mean spectra of all tissue types.
