# Supplementary material for: Optical tissue measurements of invasive carcinoma and ductal carcinoma in situ for surgical guidance
Source: Breast Cancer Res. 2021 May 22;23:59. doi: 10.1186/s13058-021-01436-5 (PMC8141169; doi:10.1186/s13058-021-01436-5)
Supplement: Supplementary file 7 — Additional file 7. Results of the GEE analysis of all spectral features. The table lists all p-values of the spectral features from the GEE-analysis. [file 13058_2021_1436_MOESM7_ESM.docx]

## Additional file 7

| Feature | p-value | | |
| --- | --- | --- | --- |
|  | IC vs DCIS | IC vs healthy | DCIS vs healthy |
| *Slopes* |  |  |  |
| 850-1122nm | 0.40 | <0.05 | <0.05 |
| 850-1210nm | 0.42 | <0.05 | <0.05 |
| 850-1350nm | 0.35 | <0.05 | <0.05 |
| 850-1440nm | 0.39 | <0.05 | 0.06 |
| 850-1584nm | 0.37 | <0.05 | <0.05 |
| 850-1598nm | 0.37 | <0.05 | <0.05 |
| 861-907nm | 0.46 | <0.05 | <0.05 |
| 870-1084nm | 0.45 | <0.05 | <0.05 |
| 874-909nm | 0.37 | <0.05 | <0.05 |
| 883-1149nm | 0.40 | <0.05 | <0.05 |
| 918-1587nm | 0.37 | <0.05 | <0.05 |
| 921-1349nm | 0.34 | <0.05 | <0.05 |
| 925-1419nm | 0.39 | <0.05 | 0.09 |
| 926-1149nm | 0.39 | <0.05 | <0.05 |
| 931-1195nm | 0.41 | <0.05 | <0.05 |
| 932-967nm | 0.18 | <0.05 | <0.05 |
| 999-1034nm | <0.05 | <0.05 | <0.05 |
| 1024-1059nm | 0.42 | <0.05 | 0.68 |
| 1043-1581nm | 0.37 | <0.05 | <0.05 |
| 1051-1557nm | 0.37 | <0.05 | <0.05 |
| 1081-1346nm | 0.32 | <0.05 | <0.05 |
| 1091-1348nm | 0.32 | <0.05 | <0.05 |
| 1112-1147nm | 0.40 | <0.05 | <0.05 |
| 1121-1404nm | 0.37 | 0.05 | 0.95 |
| 1125-1160nm | 0.34 | <0.05 | 0.24 |
| 1201-1236nm | 0.39 | <0.05 | <0.05 |
| 1210-1551nm | 0.33 | <0.05 | <0.05 |
| 1210-1561nm | 0.32 | <0.05 | <0.05 |
| 1211-1517nm | 0.35 | <0.05 | <0.05 |
| 1213-1248nm | 0.36 | <0.05 | <0.05 |
| 1214-1339nm | 0.17 | <0.05 | <0.05 |
| 1299-1334nm | 0.24 | <0.05 | <0.05 |
| 1312-1347nm | 0.28 | <0.05 | <0.05 |
| 1363-1398nm | 0.48 | <0.05 | <0.05 |
| 1372-1409nm | 0.56 | <0.05 | <0.05 |
| 1395-1430nm | 0.86 | <0.05 | <0.05 |
| 1467-1502nm | 0.89 | <0.05 | <0.05 |
| 1517-1552nm | 0.95 | <0.05 | <0.05 |
| *Local minima* |  |  |  |
| maximum difference of local minimum @932nm | 0.40 | 0.27 | 0.52 |
| wl of inflection point on left side of local minimum @932nm | 0.13 | <0.05 | <0.05 |
| wl of inflection point on right side of local miminum @932nm | 0.42 | <0.05 | <0.05 |
| maximum difference of local minimum @987nm | 0.43 | 0.64 | <0.05 |
| wl of inflection point on left side of local minimum @987nm | 0.25 | <0.05 | <0.05 |
| wl of inflection point on right side of local miminum @987nm | 0.31 | <0.05 | 0.09 |
| maximum difference of local minimum @1040nm | 0.45 | 0.75 | <0.05 |
| wl of inflection point on left side of local minimum @1040nm | 0.13 | 0.22 | 0.55 |
| wl of inflection point on right side of local miminum @1040nm | 0.54 | <0.05 | <0.05 |
| maximum difference of local minimum @1205nm | 0.34 | 0.49 | 0.13 |
| wl of inflection point on left side of local minimum @1205nm | 0.36 | <0.05 | <0.05 |
| wl of inflection point on right side of local miminum @1205nm | <0.05 | <0.05 | 0.53 |
| maximum difference of local minimum @1437nm | 0.37 | <0.05 | <0.05 |
| wl of inflection point on left side of local minimum @1437nm | 0.15 | 0.61 | <0.05 |
| wl of inflection point on right side of local miminum @1437nm | 0.14 | 0.07 | <0.05 |
| maximum difference of local minimum @1461nm | 0.61 | <0.05 | <0.05 |
| wl of inflection point on left side of local minimum @1461nm | 0.06 | 0.16 | 0.31 |
| wl of inflection point on right side of local miminum @1461nm | 0.52 | <0.05 | <0.05 |
| *Local maxima* |  |  |  |
| maximum difference of local maximum @951nm | 0.39 | 0.28 | 0.46 |
| wl of inflection point on left side of local maximum @951nm | 0.24 | <0.05 | <0.05 |
| wl of inflection point on right side of local maximum @951nm | 0.22 | <0.05 | <0.05 |
| maximum difference of local maximum @1046nm | 0.45 | 0.79 | <0.05 |
| wl of inflection point on left side of local maximum @1046nm | 0.13 | <0.05 | 0.80 |
| wl of inflection point on right side of local maximum @1046nm | 0.54 | <0.05 | <0.05 |
| maximum difference of local maximum @1052nm | 0.45 | 0.86 | <0.05 |
| wl of inflection point on left side of local maximum @1052nm | 0.13 | <0.05 | 0.09 |
| wl of inflection point on right side of local maximum @1052nm | 0.54 | <0.05 | <0.05 |
| maximum difference of local maximum @1054nm | 0.45 | 0.88 | <0.05 |
| wl of inflection point on left side of local maximum @1054nm | 0.13 | <0.05 | <0.05 |
| wl of inflection point on right side of local maximum @1054nm | 0.54 | <0.05 | <0.05 |
| maximum difference of local maximum @1071nm | 0.43 | 0.91 | <0.05 |
| wl of inflection point on left side of local maximum @1071nm | 0.08 | <0.05 | <0.05 |
| wl of inflection point on right side of local maximum @1071nm | 0.65 | 0.19 | 0.23 |
| maximum difference of local maximum @1256nm | 0.40 | <0.05 | <0.05 |
| wl of inflection point on left side of local maximum @1256nm | 0.22 | 0.80 | <0.05 |
| wl of inflection point on right side of local maximum @1256nm | 0.52 | <0.05 | <0.05 |
| maximum difference of local maximum @1259nm | 0.40 | <0.05 | <0.05 |
| wl of inflection point on left side of local maximum @1259nm | 0.22 | 0.80 | <0.05 |
| wl of inflection point on right side of local maximum @1259nm | 0.52 | <0.05 | <0.05 |
| maximum difference of local maximum @1285nm | 0.40 | <0.05 | <0.05 |
| wl of inflection point on left side of local maximum @1285nm | 0.22 | 0.94 | <0.05 |
| wl of inflection point on right side of local maximum @1285nm | 0.52 | <0.05 | <0.05 |
| wl = wavelength |  |  |  |

**Additional file 7. Results of the GEE analysis of all spectral features.** Complete list of all spectral features (from the slops and local minima/maxima). For each spectral feature, the p-value for the comparison of ‘IC’ vs ‘DCIS’, ‘IC’ vs ‘healthy’, and ‘DCIS’ vs ‘healthy’ is listed.
