# Supplementary material for: Optical tissue measurements of invasive carcinoma and ductal carcinoma in situ for surgical guidance
Source: Breast Cancer Res. 2021 May 22;23:59. doi: 10.1186/s13058-021-01436-5 (PMC8141169; doi:10.1186/s13058-021-01436-5)
Supplement: Supplementary file 8 — Additional file 8. Selection of feature set with a floating search. The table lists the set of features that is selected after the floating feature search. [file 13058_2021_1436_MOESM8_ESM.docx]

## Additional file 8

| Feature |
| --- |
| *Slopes* |
| slope 850-1122nm |
| slope 850-1584nm |
| slope 874-909nm |
| slope 921-1349nm |
| slope 1043-1581nm |
| slope 1210-1551nm |
| slope 1467-1502nm |
| *Local minima* |
| maximum difference of local minimum @932nm |
| wl of inflection point on left side of local minimum @987nm |
| wl of inflection point on left side of local minimum @1205nm |
| wl of inflection point on left side of local minimum @1437nm |
| wl of inflection point on right side of local minimum @1461nm |
| *Local maxima* |
| maximum difference of local maximum @951nm |
| maximum difference of local maximum @1052nm |
| maximum difference of local maximum @1256nm |
| wl of inflection point on right side of local maximum @1285nm |
| wl = wavelength |

**Additional file 8. Selection of feature set with a floating search.** The number of spectral features was further reduced by performing a floating feature search with the features that were significantly different between healthy tissue and IC or DCIS but not significant between IC and DCIS. This floating search was repeated 100 times that resulted in 100 sets of features with a different number of features in it. The number of features in a set differed between 2 and 40. To test the performance with each number of features, classification models were developed with all possible combinations of a Thres_maxcon_ and Thres_maxfat_ ranging between 0% and 50% and one of the set of features with 2 to 40 features in it. The performance (MCC, sensitivity, and specificity) was averaged over all models and seemed to reach an optimum at approximately 16 features (results not presented). These features were further used in the remainder of this study.
